# Genetic methods in honey bee breeding

**DOI:** 10.18699/VJGB-23-44

**Published:** 2023-07

**Authors:** M.D. Kaskinova, A.M. Salikhova, L.R. Gaifullina, E.S. Saltykova

**Affiliations:** Institute of Biochemistry and Genetics – Subdivision of the Ufa Federal Research Center of the Russian Academy of Sciences, Ufa, Russia; Institute of Biochemistry and Genetics – Subdivision of the Ufa Federal Research Center of the Russian Academy of Sciences, Ufa, Russia; Institute of Biochemistry and Genetics – Subdivision of the Ufa Federal Research Center of the Russian Academy of Sciences, Ufa, Russia; Institute of Biochemistry and Genetics – Subdivision of the Ufa Federal Research Center of the Russian Academy of Sciences, Ufa, Russia

**Keywords:** Apis mellifera, tRNAleu-COII locus, microsatellites, csd gene, bee diseases, медоносная пчела, Apis mellifera, локус tRNAleu-COII, микросателлиты, ген csd, болезни пчел

## Abstract

The honey bee Apis mellifera is a rather difficult object for selection due to the peculiarities of its biology. Breeding activities in beekeeping are aimed at obtaining bee colonies with high rates of economically useful traits, such as productivity, resistance to low temperatures and diseases, hygienic behavior, oviposition of the queen, etc. With two apiaries specializing in the breeding of A. m. mellifera and A. m. carnica as examples, the application of genetic methods in the selection of honey bees is considered. The first stage of the work was subspecies identification based on the analysis of the polymorphism of the intergenic mtDNA locus tRNAleu-COII (or COI-COII) and microsatellite nuclear DNA loci Ap243, 4a110, A24, A8, A43, A113, A88, Ap049, A28. This analysis confirmed that the studied colonies correspond to the declared subspecies. In the apiary with A. m. mellifera, hybrid colonies have been identified. A method based on the analysis of polymorphisms of the tRNAleu-COII locus and microsatellite nuclear DNA loci has been developed to identify the dark forest bee A. m. mellifera and does not allow one to differentiate subspecies from C (A. m. carnica and A. m. ligustica) and O (A. m. caucasica) evolutionary lineages from each other. The second stage was the assessment of the allelic diversity of the csd gene. In the apiary containing colonies of A. m. mellifera (N = 15), 20 csd alleles were identified. In the apiary containing colonies of A. m. carnica (N = 44), 41 alleles were identified. Six alleles are shared by both apiaries. DNA diagnostics of bee diseases showed that the studied colonies are healthy. Based on the data obtained, a scheme was developed for obtaining primary material for honey bee breeding, which can subsequently be subjected to selection according to economically useful traits. In addition, the annual assessment of the allelic diversity of the csd gene will shed light on the frequency of formation of new allelic variants and other issues related to the evolution of this gene.

## Abstract (ru)

Медоносная пчела Apis mellifera является довольно сложным объектом для селекции в силу особенностей ее биологии. Селекционные мероприятия в пчеловодстве нацелены на получение семей пчел с высокими показателями хозяйственно полезных признаков, таких как продуктивность, устойчивость к низким температурам и заболеваниям, гигиеническое поведение, яйценоскость матки и др. На примере двух пасек, специализирующихся на разведении A. m. mellifera и A. m. carnica, рассмотрено применение генетических методов в селекции медоносной пчелы. Первым этапом работы было установление подвидовой принадлежности на основе оценки полиморфизма межгенного локуса мтДНК tRNAleu-COII и микросателлитных локусов ядерной ДНК Ap243, 4a110, А24, A8, A43, A113, A88, Ap049 и A28. Подтверждено, что исследуемые семьи соответствуют заявленным подвидам. На пасеке с A. m. mellifera выявлены гибридные семьи. Метод, основанный на анализе полиморфизма локуса tRNAleu- COII (или COI-COII) и микросателлитных локусов ядерной ДНК, был разработан для идентификации темной лесной пчелы A. m. mellifera и не позволяет дифференцировать представителей эволюционных ветвей С (A. m. carnica и A. m. ligustica) и О (A. m. caucasica). На втором этапе оценивалось аллельное разнообразие гена csd. На пасеке, содержащей семьи A. m. mellifera (N = 15), выявлено 20 аллелей csd, на пасеке, содержащей семьи A. m. carnica (N = 44), – 41 аллель. Шесть аллелей были общими для двух пасек. ДНК-диагностика заболеваний пчелы показала, что исследуемые семьи являются здоровыми. По результатам исследований разработана схема получения первичного материала для селекции медоносной пчелы, на который впоследствии может быть наложен отбор по хозяйственно полезным признакам. Кроме того, ежегодная оценка аллельного разнообразия гена csd позволит пролить свет на частоту формирования новых аллельных вариантов и другие вопросы, связанные с эволюцией этого ген

## Introduction

In 1996, in the Laboratory of Biochemistry of Insect Adaptability of the Institute of Biochemistry and Genetics of the Ufa Scientific Center of the Russian Academy of Sciences, under the guidance of Professor Aleksey Gennadievich Nikolenko, studies of the honey bee were initiated (Nikonorov et al., 1998; Nikolenko, Poskryakov, 2002). This object was not chosen by chance. The most extensive habitat of the dark forest bee Apis mellifera mellifera has remained on the territory of the Republic of Bashkortostan (RB). This subspecies is native to the territory of Russia and belongs to the M evolutionary lineage

In total, four evolutionary lineages for the honey bee were identified based on morphometric (Ruttner, 1988) and genetic (De La Rúa et al., 2009; Meixner et al., 2013; Cridland et al., 2017) data. These are evolutionary lineages M (bee subspecies of the western Mediterranean and northwestern Europe A. m. mellifera and A. m. iberiensis), A (subspecies from Africa A. m. scutellata, A. m. sahariensis, etc.), C (subspecies from southeastern Europe A. m. ligustica, A. m. carnica, etc.) and O (subspecies from the Middle East and Western Asia A. m. caucasica, A. m. anatolica, etc.). The import of bee colonies of other subspecies (particularly A. m. caucasica and A. m. carnica) has led to mass hybridization and the loss of the local population of honey bees in most of Russia. Therefore, the goal was to identify honey bee subspecies in order to preserve parts of scattered local populations of A. m. mellifera. The method developed by Garnery et al. (1998) was adopted. This method is based on the analysis of the polymorphism of the tRNAleu-COII intergenic mitochondrial DNA locus and makes it possible to determine the maternal line of bees. However, using this method, it was impossible to assess the influence of the drone background and identify hybrid colonies. Therefore, the search for new informative genetic markers was continued

To establish the level of hybridization of colonies, the method proposed by Solignac et al. (2003), which is based on the analysis of polymorphism of microsatellite loci, was adapted. Out of 36 microsatellite loci, those that showed the greatest differentiating ability for reference samples of A. m. mellifera, A. m. caucasica and A. m. carnica (selected in their natural habitats in Russia) were selected (Il’yasov et al., 2007; Kaskinova et al., 2022). A set of 9 microsatellite loci made it possible to differentiate the subspecies A. m. mellifera from A. m. caucasica and A. m. carnica, but not the last two subspecies from each other. Using this method, surviving populations of A. m. mellifera were found on the territory of the RB and partially in other regions of Russia (Il’yasov et al., 2007; Kaskinova et al., 2022).

At the same time, work on DNA diagnostics of honey bee diseases was underway. In 2015, studies were launched to assess the allelic diversity of the csd (complementary sex determiner) gene of the honey bee (Kaskinova et al., 2019). These studies were initiated in response to the problem of irregular brood pattern (uneven distribution of brood cells in combs) faced by beekeepers that used instrumental insemination and closed type of bee breeding. An irregular brood pattern can be caused by brood diseases and inbreeding. The first reason was excluded by us based on the results of PCR diagnostics of bee diseases. To assess the effect of inbreeding, we analyzed the allelic diversity of the csd gene. The low allelic diversity of the csd gene results in a large number of diploid drones, which are killed by worker bees. This leads to such a phenomenon as genetic irregular brood pattern (Beye et al., 2003; Zareba et al., 2017; Mroczek et al., 2022). Genetic irregular brood pattern is more common in apiaries with a closed type of breeding (isolated apiary). We analyzed the allelic diversity of the csd gene in paternal apiaries and determined which colonies are best used for instrumental insemination of queens

All these methods have found application in practical beekeeping and in combination can be used for selection of honey bees. Based on the results of more than 20 years of research, we have developed a scheme for obtaining primary material for honey bee breeding. This scheme includes the following items: (1) honey bee subspecies identification; (2) assessment of the allelic diversity of the csd gene; (3) assessment of the health status of the bee colony based on DNA diagnostics of bee diseases. On the example of two apiaries specializing in breeding A. m. mellifera and A. m. carnica, we will consider the application of genetic methods in honey bee breeding.

## Materials and methods

Sample collection. Two breeding apiaries were selected for the study. At the first apiary (hereinafter, apiary No. 1) from the Iglinsky district of the RB, which specializes in breeding A. m. mellifera, worker bees and drones from 15 colonies were selected. In this apiary, the beekeeper uses instrumental insemination and paternal colonies of different origins. At the second apiary (No. 2) from the Chishminsky district of the RB, worker bees and drones belonging to the subspecies A. m. carnica were selected from 44 colonies. There is no breeding control in this apiary, but new queens are imported every year to maintain the genetic diversity of colonies. Worker bees (3 worker bees per colony) were used for subspecies identification, since they provide more complete information about the colony genotype. All bees were collected inside the hive from brood frames

Apis mellifera subspecies identification. DNA was isolated from the thorax muscles of worker bees using the DNAExtran- 2 kit (Syntol, Moscow). The analysis of the mtDNA tRNAleu-COII intergenic locus and 9 microsatellite loci (Ap243, 4a110, A24, A8, A43, A113, A88, Ap049, A28) was used for subspecies identification. Primer sequences are presented in Table 1. The PCR mixture for 10 samples with a total volume of 150 μl included 120 μl of distilled water, 15 μl of magnesium buffer, 3 μl of DNTP mixture (concentration 10 μmol each), 5 μl of F- and R-primer (concentration of 10 pmol/μl) and 3 μl of Taq polymerase (components of the PCR mixture produced by Sintol, Moscow). PCR mode: 3 min at 94 °С, then 30 cycles with denaturation for 30 s at 94 °С, annealing for 30 s at 49 °С (for the tRNAleu-COII locus) and 55 °С (for microsatellite loci), elongation for 60 s at 72 °С and final elongation for 3 min at 72 °С. PCR products were visualized using 8 % polyacrylamide gel electrophoresis (PAAG) followed by detection in a Gel Doc™ XR+ photosystem (BioRad, USA).

**Table 1.**
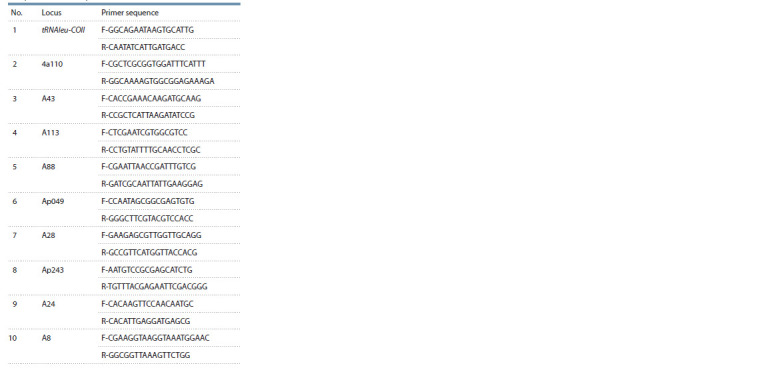
Primer sequences for Apis mellifera subspecies identification

Samples of A. m. mellifera from the Burzyansky district of the RB and Perm Krai (N = 136) were used as a reference group of the evolutionary lineage M. Samples from the Republic of Adygea, Krasnodar Krai and Uzbekistan (N = 120) were used as representatives of the C/O evolutionary lineages.

Cluster analysis was carried out using Structure 2.3.4 software. The Admixture model was used with Burnin Period and MCMC equal to 10,000 and 100,000, respectively. The number of clusters was set from 1 to 10. The estimated number of clusters was calculated using the Structure Harvester online service (Earl, von Holdt, 2012). The results obtained in Structure were processed in CLUMPP 1.1.2 using the FullSearch algorithm.

Assessment of allelic diversity of the csd gene. DNA was isolated from the thorax muscles of drones using the DNAExtran- 2 kit (Syntol, Moscow). Primers F-GGGAGAGAAG TTGCAGTAGAG and R-TTGATGCGTAGGTCCAAATCC flanking exons 6–8 of the csd gene were used (Kaskinova et al., 2019). Sequencing of PCR products was carried out at Syntol (Moscow) with forward and reverse primers used for PCR. The resulting nucleotide sequences were edited in Chromas v. 2.22 and aligned with ClustalW in MEGA v6.0. Alignment was performed on the reference sequence (NCBI Reference Sequence: NC_007072.3). Exons were identified using BLAST (https://blast.ncbi.nlm.nih.gov/Blast.cgi). The nucleotide sequences have been uploaded to Genbank and are available under numbers KY502199–KY502249 and MK531891–MK531969.

DNA diagnostics of honey bee diseases. We performed DNA diagnostics of the most common honey bee pathogens (Table 2): the fungus Ascosphaera apis, which causes ascospherosis (AscoA); microsporidium Nosema/Vairimorpha apis (NosA) and Nosema/Vairimorpha cerana (NosC), causing type A and C nosematosis, respectively; bacteria Paenibacillus larvae (AFB) and Melissococcus plutonius (EFB) causing foulbrood diseases. PCR diagnostics of bees for the presence of viral diseases was also performed: sacbrood virus (SBV), black queen cell virus (BQCV), wing deformation virus (DWV), Kashmir bee virus (KBV), acute bee paralysis virus (ABPV), chronic bee paralysis virus (CBPV) (see primer list in Table 2). DNA samples from diseased bees and synthetic DNA fragments that completely matched the expected PCR product were used as a positive control.

**Table 2.**
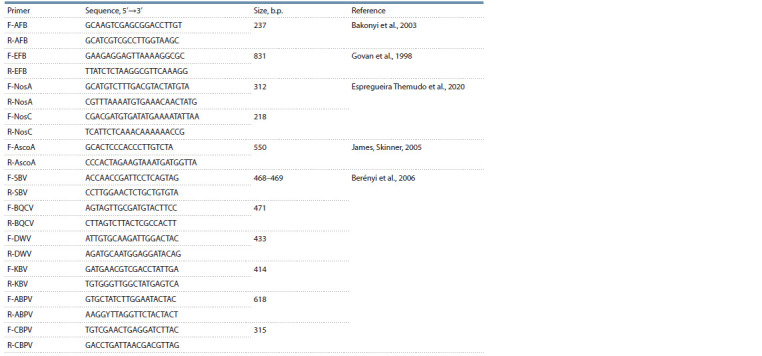
Primers for DNA diagnostics of honey bee diseases

DNA isolation was performed from the midgut of worker bees (10 worker bees from each colony) using the DNAExtran- 2 kit. The PCR mixture for 10 samples with a total volume of 150 μl included 120 μl of distilled water, 15 μl of magnesium buffer, 3 μl of DNTP mixture (concentration 10 μmol each), 5 μl of F- and R-primer (concentration of 10 pmol/μl) and 3 μl of Taq polymerase. PCR mode: 5 min at 94 °C, then 30 cycles with 30 s denaturation at 94 °C, 30 s annealing at 50 °C, 60 s elongation at 72 °C and final elongation 7 min at 72 °C. Amplification products were visualized in 8 % PAAG.

To diagnose honey bee viruses, RNA was isolated from the thorax muscles of live bees (from one colony of 20 bees frozen in liquid nitrogen, taking into account two repetitions) using Trizol (Thermo FS). For cDNA synthesis, an RT-PCR kit (Syntol, Moscow) was used. The resulting cDNA was used for further PCR.

## Results and discussion

Apis mellifera subspecies identification

Using the polymorphism analysis of the mtDNA tRNAleu- COII intergenic locus and 9 microsatellite loci, we analyzed the genetic structure of the studied samples.

Analysis of the mtDNA tRNAleu-COII intergenic locus is one of the simple and reliable methods for differentiating M and C/O evolutionary lineages. Allelic variants P(Q)1–n are markers of the origin of bees from A. m. mellifera, allelic variant Q – from subspecies from the evolutionary lineages C and O on the maternal line. P and Q are conventional designations for non-coding repeats located between the tRNAleu and COII genes. At the same time, the subspecies from the evolutionary branches C and O lack the P repeat (Bertrand et al., 2015). This analysis confirmed that the studied colonies correspond to the declared evolutionary lineages. In apiary No. 1, 14 out of 15 colonies had a PQQ allelic variant, and in one colony an allelic variant PQQQ was detected. All colonies from apiary No. 2 had the allelic variant Q.

**Fig. 1.**
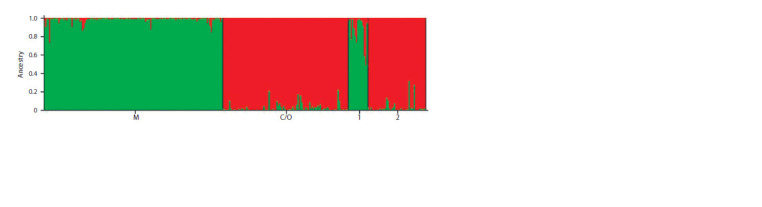
Genetic structure of the studied samples at K=2, where M is a reference sample from the M evolutionary lineage; C/O is a reference sample from the C/O evolutionary lineages; 1 is a sample from apiary No. 1; 2 is a sample from apiary No. 2.

Figure 1 shows the results of cluster analysis. Analysis of Structure output data in Structure Harvester showed that the total study sample consists of two main clusters (K = 2, delta K = 2554.6). The first cluster is represented by a dark forest bee; the second cluster includes subspecies from the evolutionary lineages C (A. m. carnica) and O (A. m. caucasica). The colonies from apiary No. 1 entered the same cluster with bees from the reference sample M (i. e., subspecies A. m. mellifera from the evolutionary lineage M). At the same time, introgression of the C/O gene pool is noted in two colonies. Colonies from apiary No. 2 form a common cluster with a C/O reference sample.

This method was developed for the identification of the dark forest bee A. m. mellifera and does not allow one to differentiate representatives of the C and O evolutionary lineages from each other. Therefore, we can say that the colonies in apiary No. 1 belong to the subspecies A. m. mellifera. Some of the colonies turned out to be hybrid and it was recommended to take measures to replace the queens. In apiary No. 2, hybrid colonies were also identified (with the share of cluster M > 15 %).

The problem of subspecies identification of the honey bee arose in connection with the uncontrolled transportation of bee colonies and the development of package beekeeping. If the differentiation of subspecies from the M and C/O evolutionary lineages is a resolved issue and is used to search for native populations (Nikolenko, Poskryakov, 2002; Il’yasov et al., 2007), then the differentiation of subspecies belonging to the same evolutionary lineage remains relevant.

Assessment of allelic diversity of the csd gene

Determining the allelic diversity of a sex-determining gene is one of the main tasks of a modern bee breeding program (Hyink et al., 2013). Keeping honey bees under closed breeding conditions results in the accumulation of a large number of common csd alleles, and consequently leads to the production of a large number of diploid drones. Diploid drones are killed by worker bees and this leads to a decrease in the strength of the colony

In apiary No. 1, 20 csd alleles described by us earlier (Kaskinova et al., 2019) were detected, in apiary No. 2 – 41 alleles. Six alleles are shared by two apiaries. Almost half of the alleles are rare, i. e. meet once (Table 3).

**Table 3.**
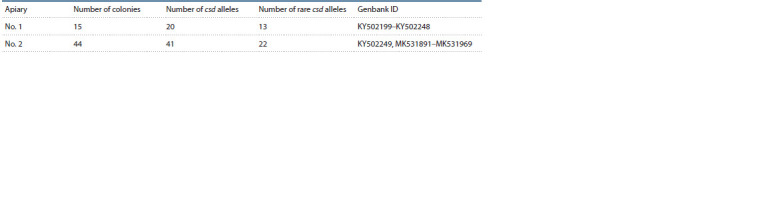
Summary table of csd alleles in the studied samples

In apiary No. 1, common alleles were identified in seven colonies (in colonies 2 and 7; 5 and 9; 5, 8 and 14, see Suppl. Material 1)1. The remaining alleles are rare. To avoid the appearance of diploid drones, it was recommended not to use together the sperm of drones from colonies with the same csd alleles during artificial insemination of queens. In apiary No. 2, almost half of the csd alleles are rare. Allele 3 csd (Suppl. Material 2) was found in 9 colonies, allele 6 – in 8 colonies. Common alleles have been identified in sister colonies descending from one queen.

Thus, we have established that nine colonies from apiary No. 1 belong to the A. m. mellifera subspecies and have a high allelic diversity of the csd gene, and therefore are recommended for further use as paternal colonies (see Suppl. Material 1). In apiary No. 2, in contrast to apiary No. 1, artificial insemination of queens is not used. Since the colonies of this apiary have a homogeneous genetic structure, colonies with the same alleles can be used as finisher colonies or a queen replacement can be carried out in them (see Suppl. Material 2). It is also possible to use these colonies as producers of honey products, preventing drones from breeding by installing drone cages.

Assessment of the health of bee colonies

PCR analysis showed that the studied colonies are healthy. Unfortunately, such healthy apiaries are the exception rather than the rule. In recent times, there has been a sad trend of a large number of apiaries suffering from nosematosis (our data, unpublished). The honey bee A. mellifera has been targeted by the dangerous invasive microsporidia species Nosema/Vairimorpha ceranae (Martín-Hernández et al., 2007; Tokarev et al., 2020), whose original host is the Asian bee Apis cerana. If earlier it was very rare for us to detect spores and DNA of this pathogen, now it is detected in almost every second apiary. This disease has no pronounced symptoms, and beekeepers seek help only when colonies begin to die or have already died. Over the entire period of the laboratory’s work, 1 Supplementary Materials 1 and 2 are available in the online version of the paper: http://vavilov.elpub.ru/jour/manager/files/Suppl_Kaskinova_Engl_27_4.pdf we have identified colonies affected by nosematosis, ascospherosis, European foulbrood, as well as such viral diseases as sacbrood virus (SBV), wing deformation virus (DWV) and black queen cell virus (BQCV). As a rule, most of these colonies were imported from other countries and belong to the evolutionary branches C or O.

Supplementary Materials are available in the online version of the paper: http://vavilov.elpub.ru/jour/manager/files/Suppl_Kaskinova_Engl_27_4.pdf

Scheme for obtaining primary material for bee breeding

Honey bee breeding is aimed at obtaining bee colonies with high rates of economic useful traits (EUT), such as productivity, resistance to low temperatures and diseases, hygienic behavior, queen egg production, etc. (Ruttner, 1988). The honey bee is a rather difficult object for selection due to the peculiarities of its biology. Complementary sex determination, in which sex depends on the allelic combination of the csd gene, enhances the consequences of inbreeding, and queen polyandry complicates the selection of parental pairs and reproduction control. In addition, the selection is carried out not at the individual level, but at the level of the bee colony. In this regard, there was a need to develop a method that would combine the assessment of EUT and the genetic potential of bee colonies. The assessment of EUT is entirely up to the beekeepers, while the result of our study was the method of selecting primary material for selection for EUT.

So-called ‘purebred breeding’ is a fundamental principle of bee breeding, according to Ruttner (1988). Based on this principle, we have developed a scheme for obtaining primary material for honey bee selection

The first stage of selection work is the selection of colonies from the initial population of bees belonging to one subspecies. In hybrid colonies, it is necessary to carry out measures to replace queens. In addition, selected colonies are encouraged to be checked for diseases

The second stage is the selection of colonies by EUT. Selected colonies are recommended to be placed in an apiary remote from other colonies from the original population in order to avoid unwanted crossbreeding. It is recommended to create several such apiaries for their further crossing. This step is optional if the goal is to restore the A. m. mellifera population.

At the third stage, the analysis of the allelic diversity of the csd gene in the selected colonies is carried out. Based on the data on the allelic composition of the csd gene, paternal and maternal colonies are formed. Maternal and paternal colonies should have different csd alleles. As maternal it is recommended to use colonies that have frequently occurring csd alleles. Paternal colonies are best formed from among those colonies that have rare csd alleles

At the fourth stage, the offspring are evaluated for EUT. Selection for the selected trait(s) must be carried out in each generation.

**Fig. 2.**
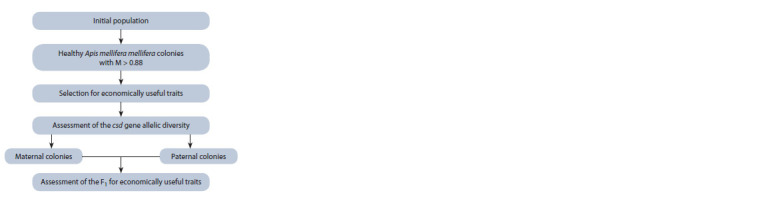
Selection scheme for the Apis mellifera mellifera using information on subspecies and allelic diversity of the csd gene.

Such a selection scheme (Fig. 2) will make it possible to obtain lines of bees with high productivity rates. In addition, the annual assessment of the allelic diversity of the csd gene in one apiary will shed light on the frequency of formation of new allelic variants and other issues related to the evolution of this gene

Analysis of the polymorphism of the sex-determining gene in the studied samples of A. mellifera generally showed that their allelic diversity corresponds to previously obtained data for other populations (Hyink et al., 2013; Zareba et al., 2017). Most bee colonies living in a breeding apiary have many common csd alleles. Over time, this can lead to large numbers of diploid drones. Therefore, it is recommended to take measures to prevent the use of drones with the same csd alleles. Moreover, it is more important that the csd alleles of the drones do not coincide with the alleles of the queen.

## Conclusion

The purpose of this work was to present the results of more than 20 years of work aimed at finding and preserving the native population of the dark forest bee A. m. mellifera. During this time, methods have been developed for the subspecies identification of A. m. mellifera, DNA diagnostics of diseases and assessment of the allelic diversity of the csd gene. These methods were tested in two apiaries using different breeding subspecies and different methods of breeding control. With the help of genetic methods, it became possible to obtain primary material for selection of honey bees, which can subsequently be subjected to selection for economically useful traits.

The search for surviving native (or local) honey bee populations is only the first step in their conservation. Further actions are completely dependent on the beekeepers – whether they will use the data received or leave everything as it is. We received positive feedback from beekeepers from the Yanaulsky, Burzyansky and Iglinsky districts of the RB, as well as from beekeepers in Altai krai, Belgorodskaya oblast and some other regions of the Russian Federation. Thanks to the initiative of beekeepers, it was possible to improve the situation even in the moderately hybrid population of A. m. mellifera in the Iglinsky district of the RB (Kaskinova et al., 2022)

Without the interest of beekeepers in the conservation of native subspecies (be it A. m. mellifera, A. m. carnica, A. m. caucasica or any other subspecies), the results of monitoring the subspecies of bees will be important only for fundamental science. Thus, based on the geographical distribution of monitoring data for the Burzyan population of A. m. mellifera, the central, peripheral, and hybrid zones of the range were identified, as well as the main directions of introgression of subspecies from C/O evolutionary lineages (Nikolenko et al., 2010). The subsequent analysis of bees throughout the territory of the RB showed that a similar subdivision of the honey bee population is also observed in other surviving populations of A. m. mellifera (Tatyshlinsky and Yanaulsky populations)

The honey bee is a very valuable and fragile object of our ecosystem. Its numbers are decreasing all over the world due to the uncontrolled use of pesticides and insecticides, hybridization, and the spread of diseases (Neumann, Carreck, 2010; Espregueira Themudo et al., 2020). Human intervention in the process of its natural settlement has practically nullified the adaptive potential that was developed over millions of years of evolution. Therefore, in our opinion, it is necessary to preserve and, if possible, restore those populations of honey bees that still remain.

Dedicated to the blessed memory of our colleagues Nikolenko Alexei Gennadievich and Poskryakov Alexander Vitalievich.

## Conflict of interest

The authors declare no conflict of interest.
